# JARID2 and the PRC2 complex regulate skeletal muscle differentiation through regulation of canonical Wnt signaling

**DOI:** 10.1186/s13072-018-0217-x

**Published:** 2018-08-17

**Authors:** Abhinav Adhikari, Judith Davie

**Affiliations:** Department of Biochemistry and Molecular Biology and Simmons Cancer Institute, Southern Illinois University School of Medicine, Carbondale, IL 62901 USA

**Keywords:** jarid2, Ezh2, Wnt, Sfrp1, MyoD

## Abstract

**Background:**

JARID2 is a non-catalytic member of the polycomb repressive complex 2 (PRC2), which is known to regulate developmental target genes in embryonic stem cells. Here, we provide mechanistic insight into the modulation of Wnt signaling by JARID2 during murine skeletal muscle differentiation.

**Results:**

We show that JARID2 is expressed in proliferating myoblasts, but downregulated upon muscle differentiation. Unexpectedly, depletion of JARID2 or the catalytic subunit of the PRC2 complex, EZH2, inhibited differentiation, suggesting that JARID2 and the PRC2 complex are required to initiate this process. Expression of the myogenic regulatory factors required to promote differentiation, MYOD and MYOG, was downregulated in the absence of JARID2, even though decreases in the methylation of histone H3 lysine 27 (H3K27^me3^) were observed on both promoters. We found that activation of the Wnt signaling pathway upregulated MYOD and restored differentiation. Activation of the Wnt pathway in JARID2 depleted cells caused β-catenin to translocate to the nucleus, where it bound to and activated the *Myod1* promoter. We show that the Wnt antagonist SFRP1 is highly upregulated in the absence of JARID2 and is a direct target of JARID2 and the PRC2 complex. Ectopic expression of SFRP1 blocked MYOD and late muscle gene expression and inhibited the translocation of β-catenin to the nucleus. Finally, we show that JARID2 and SFRP1 are inversely correlated in melanoma, confirming that the JARID2-mediated repression of SFRP1 extends beyond skeletal muscle and has important implications in many cellular systems, including cancer.

**Conclusions:**

We show that JARID2 and the PRC2 complex regulate muscle differentiation by modulating Wnt signaling through the direct repression of Wnt antagonists.

**Electronic supplementary material:**

The online version of this article (10.1186/s13072-018-0217-x) contains supplementary material, which is available to authorized users.

## Background

Skeletal muscle differentiation is controlled by four highly related basic helix-loop-helix proteins known as the myogenic regulatory factors (MRFs), which include MYF5, MYOD, MYOG and MYF6. The process of differentiation is a tightly regulated process that is modulated by many signaling pathways, including the Wnt signaling pathway. Wnt functions through both a canonical and non-canonical pathway, and both contribute to skeletal muscle growth and homeostasis [1]. In the canonical pathway, binding of Wnt proteins to the frizzled (FZD) receptor activates β-catenin, which is normally associated with its own degradation complex that includes the serine–threonine kinase glycogen synthase kinase 3 (GSK3), AXIN and adenomatous polyposis coli (APC). Phosphorylation of β-catenin by GSK3 within the destruction complex leads to its ubiquitin-mediated proteasomal degradation. However, binding of Wnt ligand to FZD receptors activates disheveled (DSH) and heterotrimeric G proteins that lead to recruitment of AXIN to the intracellular domain of the FZD co-receptor, low-density lipoprotein receptor-related protein (LRP). This leads to disassembly of the destruction complex releasing free β-catenin in the cytoplasm, which later translocates to the nucleus and activates gene expression in concert with the T cell factor (TCF) and lymphoid enhancer-binding factor (LEF) families of transcription factors. One of the targets of β-catenin has been shown to be *Myod1* [2].

Polycomb repressor proteins have important developmental roles and play essential roles in skeletal muscle specification. The two major polycomb complexes, polycomb repressive complex 1 (PRC1) and polycomb repressive complex 2 (PRC2), are responsible for the ubiquitination of histone H2A on lysine 119 (H2AK119) and the methylation of H3 lysine 27 (H3K27), respectively [3–5]. Methylation of H3K27 recruits PRC1, which then catalyzes the ubiquitination of H2AK119. The methylation of H3K27 by PRC2 requires the presence of one of the enhancer of Zeste proteins, EZH1 or EZH2. Di- and trimethylation of H3K27 is primarily associated with facultative heterochromatin, while mono-methylation is associated with intergenic heterochromatin. Promoters and enhancers of many lineage-specific genes contain di- and trimethylated H3K27, and many of these elements lose the methylation profile upon differentiation (reviewed in [6]). In skeletal muscle, EZH2 is co-expressed with PAX7, which is essential for satellite cell specification and maintenance [7] and appears to be required for homeostasis of the satellite cell pool [8]. Consistent with this role, EZH2 expression is downregulated with the differentiation of myoblasts and the activation of muscle gene expression [9].

JARID2 is the catalytically inactive founding member of the jumonji family of proteins. JARID2 is a sub-stoichiometric component of PRC2 that appears to optimize the enzymatic activity of PRC2 and is thought to function in recruiting PRC2 [10]. JARID2 is known to be involved in targeting the PRC2 complex, but how JARID2 recognizes target sites is unclear. We have shown that JARID2 is recruited by interferon-gamma-stimulated CIITA to repress muscle-specific genes [11]. In embryonic stem (ES) cells, deletion of *Jarid2* shows a severe differentiation block due to an upregulation of *Nanog* mRNA and an inhibition of Wnt signaling [12]. ChIP sequencing data for JARID2 in ES cells showed JARID2 bound to 1337 promoters, including genes critical for Wnt signaling and Wnt-related pathways [13]. Included in this group were two Wnt antagonists, secreted frizzled related protein 1 (*Sfrp1*) and naked cuticle homolog 1 (*Nkd1*). *Sfrp1* is one of five highly related proteins in mouse and human that encode secreted Wnt antagonists [14–16], and *Nkd1* encodes a disheveled binding protein that acts as a negative regulator of Wnt [17]. *Sfrp1* was one of a panel of Wnt signaling genes shown to be upregulated in embryonic stem (ES) cells upon JARID2 deletion [12].

To understand the role of JARID2 in skeletal muscle more clearly, we sought to understand the expression and function of JARID2 during muscle differentiation in this work. We show that, as previously shown for EZH2, JARID2 is downregulated upon skeletal muscle differentiation. We anticipated that depletion of JARID2 and EZH2 would promote differentiation, but unexpectedly, we found that JARID2 and EZH2 were instead required to initiate differentiation. Depletion of *Jarid2* mRNA blocked the expression of MYOD and MYOG. We found that restoration of Wnt signaling restored MYOD expression and muscle differentiation, and we show that JARID2 and the PRC2 complex directly repress an antagonist of the Wnt signaling pathway, *Sfrp1*. Thus, we show the essential role that JARID2 and the PRC2 complex play in the differentiation of skeletal muscle and establish that they act to modulate Wnt signaling in muscle. Finally, we show that JARID2 and SFRP1 are negatively correlated in cancer and correlate with overall survival and life span, suggesting that the repression of SFRP1 by JARID2 has important implications outside of skeletal muscle as well.

## Results

**Fig. 1:**
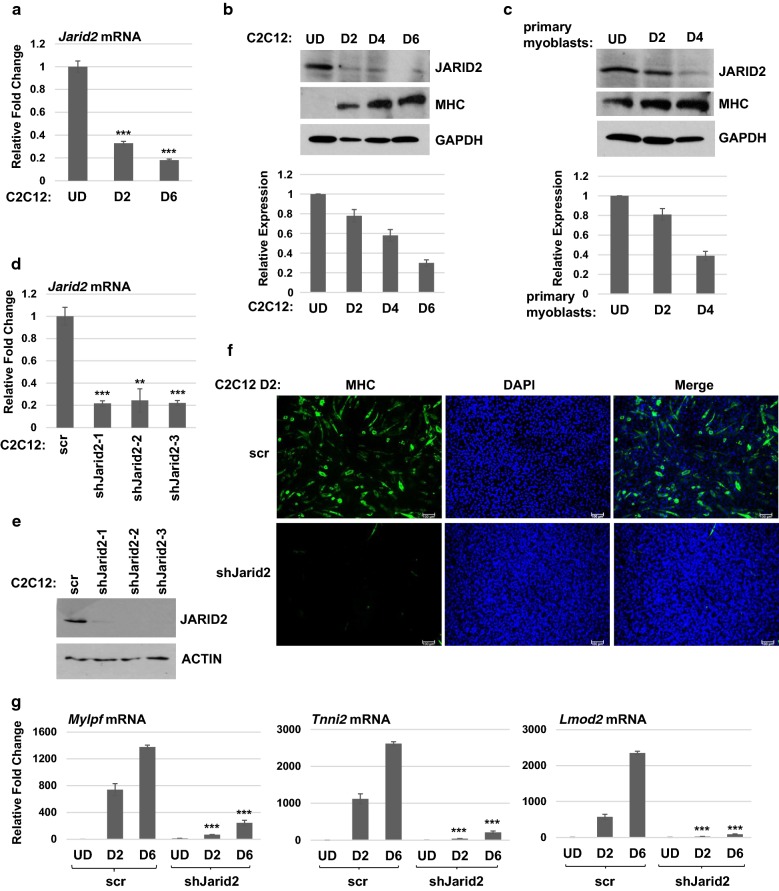
JARID2 expression is downregulated upon differentiation in C2C12 cells, but is required for differentiation. **a** Time-course analysis of *Jarid2* mRNA expression in C2C12 cells analyzed by qRT-PCR. UD represents undifferentiated and D2, D6 represent the days of differentiation. Error bars are S.E.M. ***p value < 0.001 versus UD. *n* ≥ 3. **b** Time course of JARID2 and MHC protein expression in C2C12 cells analyzed by western blot assays. JARID2 expression was quantitated with respect to the normalizer (GAPDH) in the lower panel. Error bars are S.E.M. *n* ≥ 3. **c** Time course of JARID2 and MHC expression in primary myoblasts analyzed as in **b**. **d** *Jarid2* mRNA was depleted in C2C12 cells using stable cell lines expressing three individual shRNA constructs. Depletion was confirmed by qRT-PCR analysis of mRNA. Scr represents the scrambled control, and shJarid2 represents shRNA against *Jarid2* mRNA. Error bars are S.E.M. **p value < 0.01 and ***p value < 0.001 versus scr. *n* ≥ 3. **e** Depletion of JARID2 protein was confirmed by western blot assays. **f** Differentiation is impaired upon loss of JARID2. Cells were differentiated for 2 days (D2) for immunohistochemistry with antibodies against myosin heavy chain (MHC). Nuclei were stained with DAPI. Images were taken at × 400 magnification. Bars represent 100 μm. **g** mRNA of differentiation-specific genes as indicated were downregulated in JARID2 depleted cells as assayed by qRT-PCR. Time points are indicated as in **a**. Error bars are S.E.M. ***p value < 0.001 versus time-matched scr. *n* = 3

To establish the function of JARID2 in skeletal muscle, we first sought to understand the expression of JARID2 in a time course of muscle differentiation using C2C12 cells, an immortalized murine cell line model of myogenesis. We found that *Jarid2* mRNA was expressed in proliferating myoblasts, but was downregulated upon muscle differentiation (Fig. 1a). Similar results were observed for the protein expression of JARID2 (Fig. 1b). The expression of myosin heavy chain (MHC) was used as a marker of differentiation. To confirm that these results reflected events occurring in normal skeletal muscle, we repeated the experiment using freshly derived primary myoblasts. We found that JARID2 was downregulated upon differentiation in these cells as well (Fig. 1c). To determine whether JARID2 influenced myogenesis, we depleted *Jarid2* mRNA with a series of shRNA constructs in C2C12 cells. The depletion of *Jarid2* mRNA by each shRNA construct was confirmed at the level of RNA (Fig. 1d) and protein (Fig. 1e). We selected the shRNA constructs shJarid2-1 and shJarid2-3 for further analysis. The results from both lines were similar, so only the results from shJarid2–3 are further reported here. When the cell lines were subjected to differentiation conditions, we found that differentiation was strongly inhibited in the JARID2 depleted cell line as confirmed by immunohistochemistry for MHC (Fig. 1f). Additionally, RNA analysis for the expression of a three differentiation-specific markers; myosin light chain phosphorylatable, fast (*Mylpf*), troponin I, skeletal, fast 2 (*Tnni2*) and leiomodin 2 (*Lmod2*) confirmed the expression block to differentiation-specific genes (Fig. 1g).

**Fig. 2:**
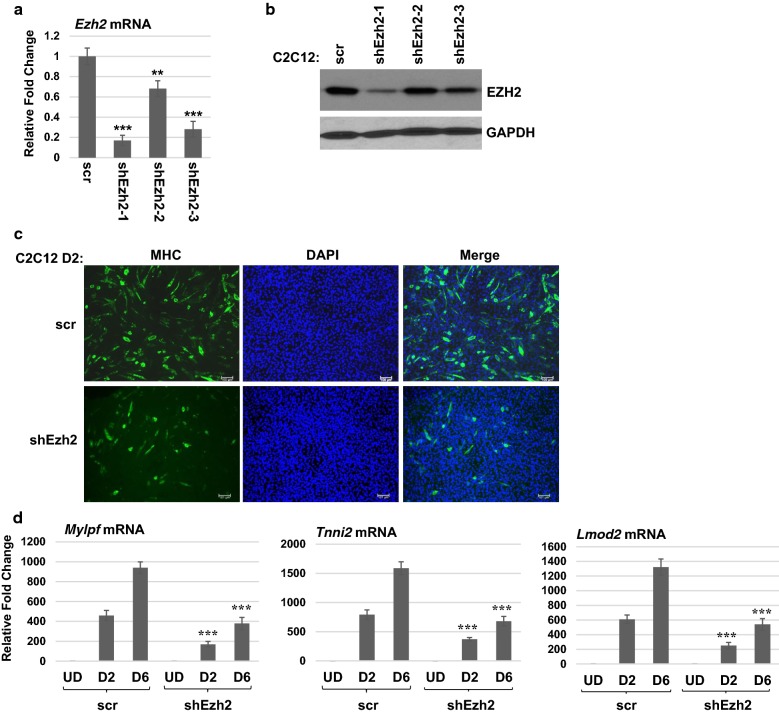
Loss of EZH2 also inhibits C2C12 differentiation. **a** *Ezh2* mRNA was depleted using stable cell lines expressing control scramble shRNA (scr) or individual *Ezh2*-specific shRNA (shEzh2) constructs. Depletion was confirmed by analysis of mRNA by qRT-PCR. Error bars are S.E.M. **p value < 0.01 and ***p value < 0.001 versus scr. *n* ≥ 3. **b** EZH2 protein depletion was confirmed by western blot assays. **c** Differentiation is impaired upon EZH2 depletion. Cells were differentiated for 2 days for immunohistochemistry with antibodies against myosin heavy chain (MHC). Nuclei were stained with DAPI. Images were taken at × 400 magnification. Bars represent 100 μm. **d** mRNA of differentiation-specific genes as indicated was downregulated in EZH2 depleted cells assayed by qRT-PCR. Error bars are S.E.M. **p value < 0.01 versus time-matched scr. *n* ≥ 3

JARID2 is a non-catalytic member of the PRC2 complex, so we next asked whether this effect was a result of the loss of JARID2, or a loss of PRC2 complex activity. We did this by depleting the catalytic subunit of PRC2, EZH2, using shRNA constructs. We identified a shRNA, shEzh2-1, that led to a significant depletion of *Ezh2* mRNA that could be observed at the level of RNA (Fig. 2a) and protein (Fig. 2b). When subjected to differentiation conditions, the EZH2 depleted cell line also showed an inhibition of differentiation as assayed by MHC immunohistochemistry (Fig. 2c). RNA analysis also showed a significant reduction in the expression of *Mylpf*, *Tnni2* and *Lmod2* (Fig. 2d). The block to differentiation is not as robust as observed for JARID2 depleted lines, but JARID2 was also depleted more efficiently than was EZH2, complicating any comparison of the effects in the two cell lines. However, the data clearly show that both JARID2 and EZH2 are required to initiate differentiation.

**Fig. 3:**
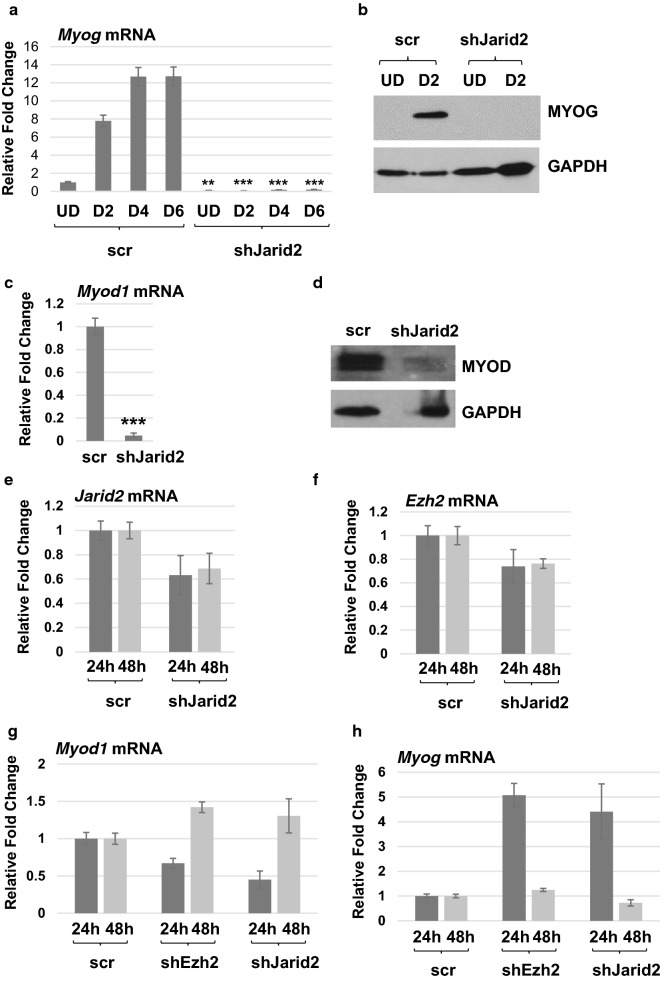
JARID2 is required for the expression of myogenin and MyoD. **a** Myogenin expression is downregulated in JARID2 depleted C2C12 cells. mRNA was assayed in C2C12 cells stably expressing scramble control (scr) and shRNA against *Jarid2* mRNA (shJarid2). qRT-PCR was performed on undifferentiated (UD) and 2, 4, 6 days of differentiation (D2, D4, D6). Error bars are S.E.M. **p value < 0.01 and ***p value < 0.001 versus time-matched scr. *n* ≥ 3. **b** MYOG is downregulated upon JARID2 depletion as detected by western blot assays. **c** mRNA expression of *Myod1* is downregulated in undifferentiated JARID2 depleted cells as assayed by qRT-PCR. Error bars are S.E.M. ***p value < 0.001 versus scr. *n* ≥ 3. **d** Protein expression of MYOD is downregulated in JARID2 depleted cells as assayed by western blot assays. ***p value < 0.001. *n* ≥ 4. e shJarid2 constructs were transiently transfected into C2C12 cells and assayed for *Jarid2* mRNA expression 24 and 48 h post-transfection. **f** shEzh2 constructs were used as in **e** and assayed for *Ezh2* mRNA expression. **g**, **h** shJarid2 and shEzh2 transient transfections were assayed for *Myod1* (**g**) and *Myog* (**h**) mRNA expression. Error bars are S.E.M. *n* ≥ 3

This requirement was very surprising, as we had anticipated that the loss of the PRC2 complex would enhance differentiation. Many differentiation-specific genes are controlled by the chromatin target of PRC2, methylation of histone H3 lysine 27 (H3K27^me^) [18]. Myogenin (MYOG), the MRF required for myoblast fusion and differentiation, is known to be a target of the PRC2 complex [18]. Thus, we examined the expression of *Myog* in the JARID2 depleted line and found that MYOG was not upregulated upon differentiation in the JARID2 depleted line. This result could be observed at the level of both RNA (Fig. 3a) and protein (Fig. 3b). Next, we examined the expression of *Myod1* and found that MYOD was also strongly repressed in the JARID2 depleted line at the level of both RNA (Fig. 3c) and protein (Fig. 3d). JARID2 has been shown to be both inhibitory for PRC2 complex activity and required for PRC2 targeting, suggesting that JARID2 acts to “fine-tune” PRC2 complex activity [13, 19]. To determine how depletion of JARID2 impacted the activity of the PRC2 complex on the promoters of *Myod1* and *Myog*, we performed chromatin immunoprecipitation (ChIP) assays with antibodies against H3K27^me3^ and examined the enrichment on the *Myod1* and *Myog* promoters in the presence or depletion of JARID2. *MyoD1* is regulated by several major upstream cis-elements including the distal regulatory region (DRR), core enhancer (CE) and proximal regulatory region (PRR) [20, 21]. We found that H3K27 methylation could be observed at the DRR, CE and PRR of the *Myod1* promoter and that depletion of JARID2 leads to a loss of H3K27^me3^ on each of these regions (Additional file 1: Figure S1 a–c). H3K27 methylation was also observed on the myogenin promoter in a previously identified region 1.5-kb upstream of the transcriptional start site [18], and depletion of JARID2 also led to a reduction of H3K27^me3^ in this region (Additional file 1: Figure S1 d). No change in methylation was observed on the promoter proximal region of the myogenin promoter (Additional file 1: Figure S1 e). As a control for these experiments, we show that H3K27^me3^ is observed on the *HoxB7* promoter (Additional file 1: Figure S1 f), a known target of the PRC2 complex [18]. We show here that methylation of the *HoxB7* promoter is independent of JARID2. Significantly, we also show here that JARID2 is required to guide the PRC2-mediated H3K27 methylation responsible for repressing both *Myod1* and *Myog* and that JARID2 does not inhibit PRC2 complex activity on these promoters. In cardiac cells, JARID2 has been shown to guide histone H3 lysine 9 (H3K9^me^) methylation to target genes [22]. Thus, we also examined the enrichment of H3K9^me^ on the *Myod1* and *Myog* promoters in the presence or depletion of JARID2. We found no decrease in H3K9^me^ on the *Myod1* promoter (Additional file 1: Figure S1 g–i) or the promoter proximal region of the *Myog* promoter (Additional file 1: Figure S1 j). A reduction in H3K9^me^ was observed on the 1.5-kb upstream region of the myogenin promoter (Additional file 1: Figure S1 k), indicating that JARID2 may play a role in directing H3K9^me^ in this region. However, our data show that the loss of the repressive methylation of H3K27 or H3K9 is not sufficient for activation of the MRFs. Instead, our data suggested that JARID2 and the PRC2 complex must have an indirect role in activating *Myod1* and *Myog* upon differentiation.

These results were very surprising given the previously published work, showing that transient siRNA depletion of another component of the PRC2 complex, SUZ12, led to a precocious activation of myogenin RNA [18]. Other work has shown no change in *Myog* mRNA expression upon a transient siEZH2 depletions and a delay in *Myog* mRNA expression upon transient siSUZ12 depletion [23]. To reconcile our results to these studies, we performed transient transfections with the shJarid2 and shEzh2 constructs used to construct our stable cell lines and examined gene expression profiles 24 or 48 h post-transfection. As anticipated, *Jarid2* mRNA (Fig. 3e) and *Ezh2* mRNA (Fig. 3f) were depleted by the respective shRNA constructs. We found that *Myog* mRNA was upregulated 24 h post-transfection, but *Myog* mRNA levels were returned to normal levels by 48 h (Fig. 3h). *Myod1* mRNA was modestly downregulated after 24 h, but restored to normal or slightly elevated levels by 48 h (Fig. 3g). These results strongly suggest that the sustained depletion of *Jarid2* mRNA and *Ezh2* mRNA in our stable cell lines is required for the inhibition of MYOD and MYOG we observe.

To determine whether the loss of MYOD was responsible for the block to differentiation we observed, we asked whether ectopic expression of MYOD in JARID2 depleted cells could rescue differentiation. An expression construct for *Myod1* (cDNA) was transiently expressed in JARID2 depleted cells, and we found that, as anticipated, *Myod1* mRNA expression was observed (Additional file 2: Figure S2 a). We next examined *Myog* mRNA expression and found that expression of MYOD also upregulated *Myog* mRNA (Additional file 2: Figure S2 b). Analysis of a differentiation-specific marker, *Mylpf*, also showed upregulation upon expression of MYOD (Additional file 2: Figure S2 c). These data strongly suggested that the inhibition of *Myod1* was largely responsible for the block to differentiation in JARID2 depleted cells.

**Fig. 4:**
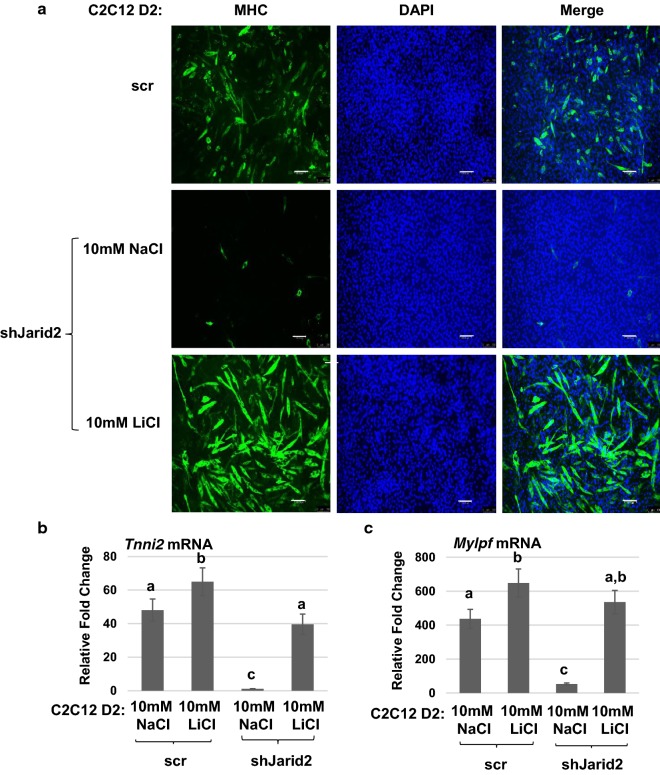
Wnt signaling is modulated by JARID2. **a** Activation of canonical Wnt signaling restores differentiation in JARID2 depleted cells. shJarid2 cells were treated with either 10 mM NaCl or 10 mM LiCl for 2 days in differentiation conditions before immunohistochemistry with myosin heavy chain (MHC) antibodies. Nuclei were stained with DAPI. Images were taken at × 400 magnification. Bars represent 100 μm. **b**, **c** Differentiation-specific gene expression is induced by Wnt activation. Expression of *Tnni2* mRNA (**b**) and *Mylpf* mRNA (**c**) was quantitated by qRT-PCR. Fold change in expression of mRNA was calculated relative to undifferentiated scramble control. Data are plotted as means with S.E.M. and analyzed with one-way ANOVA followed by Tukey’s HSD post hoc test. Means not sharing same letter are statistically significant (*p* < 0.001). *n* ≥ 4

The regulation of *Myod1* is complex and impacted by many signaling pathways, including the Wnt signaling pathway (reviewed in [1]). To determine whether modulation of Wnt signaling could impact the JARID2-mediated block to differentiation, we treated JARID2 depleted cells with a chemical inhibitor of GSK3β, lithium chloride (LiCl). Inhibition of GSK3β by LiCl is known to enhance myotube formation in C2C12 cells [24]. Treatment with NaCl was used as a nonspecific control. We found that activation of Wnt signaling by treatment with LiCl completely restored differentiation in JARID2-depleted cells as assayed by immunohistochemistry for MHC (Fig. 4a). RNA analysis also showed a restoration of differentiation-specific gene expression including *Tnni2* mRNA (Fig. 4b) and *Mylpf* mRNA (Fig. 4c).

**Fig. 5:**
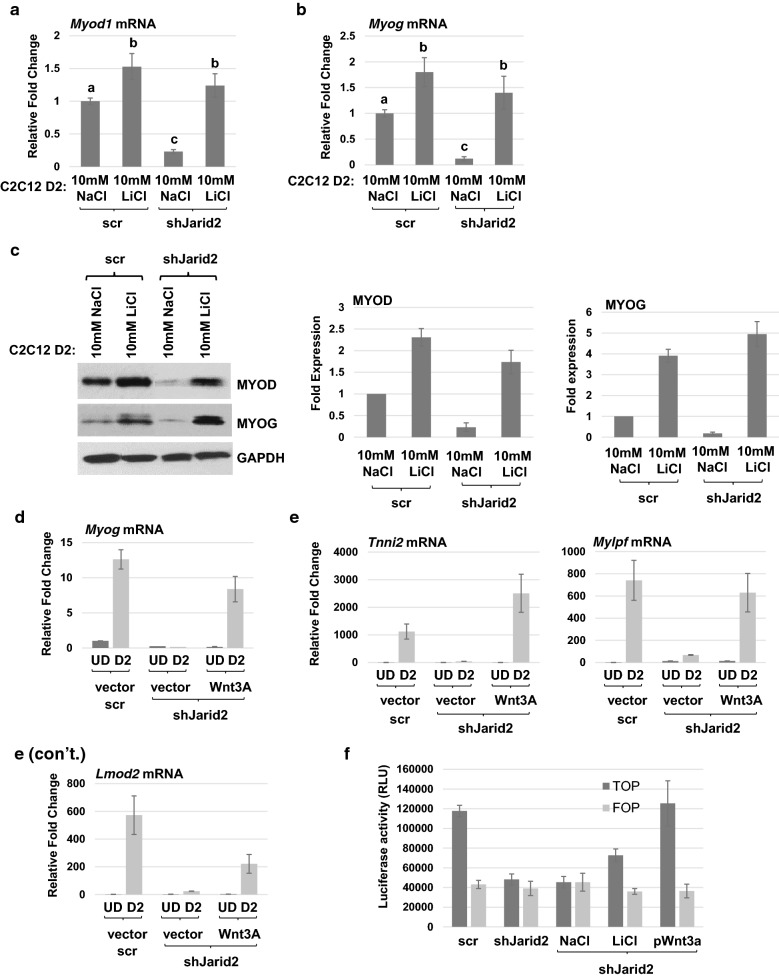
Activation of canonical Wnt signaling rescues MRF expression. **a**, **b** Scr- or shJarid2-expressing cells were differentiated for 2 days (D2) and shJarid2 cells were treated with either 10 mM NaCl or 10 mM LiCl while differentiating. *Myod1* (**a**) and *Myog* (**b**) mRNA expressions were analyzed by qRT-PCR. Fold change in expression of mRNA was calculated relative to 10-mM NaCl-treated scr control (D2). Data are plotted as means with S.E.M. and analyzed with one-way ANOVA followed by Tukey’s HSD post hoc test. Means not sharing same letter are statistically significant (*p* < 0.001). *n* ≥ 4. **c** Cells as in **a** were analyzed for protein expression of MYOD and MYOG by western blot assays. Data are plotted as bar graphs with respect to the normalizer (GAPDH) in the right panel. Error bars are S.E.M. *n* ≥ 3. **d**, **e** Scr- or shJarid2-expressing cells were transiently transfected with an expression construct for *Wnt3a* (cDNA) or vector control and assayed for mRNA expression of *Myog* (**d**) and differentiation-specific genes (**e**) by qRT-PCR. UD represents undifferentiated myoblasts, and D2 represents 2 days of differentiation. Fold change was calculated relative to scr UD. Error bars are S.E.M. *n* ≥ 3. **f** JARID2 depletion impairs Wnt-mediated transcriptional activity and can be rescued by LiCl or WNT3A. Reporter assays were performed using TOP*Flash* and FOP*Flash* luciferase reporters in scr or shJarid2 cells. A set of shJarid2 cells was also transfected with an expression construct for *Wnt3a* (cDNA). Growth medium was replaced with differentiation medium with or without LiCl or NaCl 24 h post-transfection, and luciferase activity was detected 48 h post-transfection. Error bars are S.E.M. *n* ≥ 3

We next asked whether expression of MYOD and MYOG was restored upon activation of Wnt. We found that the mRNA expression of *Myod1* (Fig. 5a) and *Myog* (Fig. 5b) was restored at the RNA level following LiCl treatment. The activation of MYOD and MYOG was confirmed at protein expression level (Fig. 5c). We sought to confirm our results using an expression construct for *Wnt3a* (cDNA) [25]. We found that ectopic WNT3A promoted the expression of *Myog* mRNA in JARID2 depleted cells (Fig. 5d). RNA expression of the differentiation-specific markers *Mylpf*, *Tnni2* and *Lmod2* was also activated by WNT3A in JARID2-depleted cells (Fig. 5e). As an additional confirmation of the block to Wnt signaling in these cells, we also used the TOP*Flash*/FOP*Flash* luciferase reporter system. We found that TOP*Flash*, which contains intact TCF-binding sites, was more active than FOP*Flash*, which contains mutated TCF-binding sites, in scr control cells. However, in cells depleted for JARID2, TOP*Flash* was not activated. Treatment of shJarid2 cells with LiCl restored activation of TOP*Flash.* Transfection of *Wnt3a* (cDNA) also robustly restored TOP*Flash* activation (Fig. 5f). Taken together, these data strongly support a block to Wnt signaling in the absence of JARID2.

**Fig. 6:**
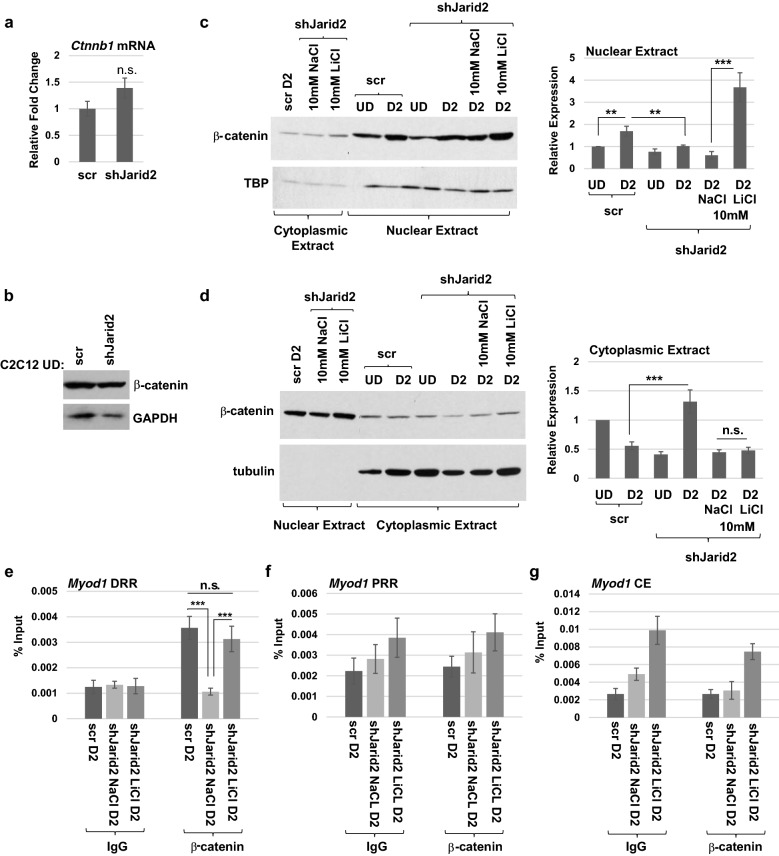
Activation of canonical Wnt signaling induces translocation of β-catenin to the nucleus in JARID2 depleted cells. **a**, **b** Expression of β-catenin is unchanged with loss of JARID2 as assayed for mRNA by qRT-PCR (**a**) and protein by western blot assays (**b**). Error bars are S.E.M. *n* ≥ 3. n.s. represents not significant statistical difference between the means. **c**, **d** Localization β-catenin is altered upon JARID2 depletion. C2C12 cells stably expressing scr or shJarid2 were grown to confluency in high serum (UD) and switched to differentiation conditions for 2 days (D2). shJarid2 cells were treated with either 10 mM NaCl or 10 mM LiCl for 2 days in differentiation conditions as indicated. Both nuclear (**c**) and cytoplasmic (**d**) fractions of proteins were extracted and probed for ß-catenin, TBP (loading control for nucleus) and TUBULIN (loading control for cytoplasm). Gels were quantified and normalized to respective loading controls (right side of panel). Relative expression was calculated relative to scr UD sample and plotted as bar graphs. Error bars are S.E.M. *n* ≥ 3. **e**–**g**. Canonical Wnt signaling directly regulates *Myod1* expression through binding of β-catenin in the distal regulatory region of the *Myod1* promoter. C2C12 cells stably expressing scramble shRNA (scr) or *Jarid2* mRNA-specific shRNA(shJarid2) were grown to confluency and switched to differentiation conditions for 2 days (D2). shJarid2 cells were treated with either 10 mM NaCl or 10 mM LiCl for 2 days in differentiation conditions. ChIP assays using antibodies against β-catenin and a nonspecific antibody (IgG) were performed using primers spanning three different regions; distal regulatory region (DRR) (**e**), proximal regulatory region (PRR) (**f**), and core enhancer (CE) (**g**) of the *Myod1* promoter. Error bars are S.E.M. ***p value < 0.001. *n* ≥ 3

To understand how Wnt signaling was blocked in JARID2-depleted cells, we examined the expression of the downstream mediator of canonical Wnt signaling, β-catenin (*Ctnnb1*). Analysis of the RNA expression level of *Ctnnb1* showed no significant deregulation (Fig. 6a). Protein expression in total cell extracts also showed no significant change of β-catenin expression in JARID2-depleted cells (Fig. 6b). While we saw no obvious change in total β-catenin expression in JARID2-depleted cells, we next asked whether β-catenin localization was altered in these cells. Nuclear and cytoplasmic fractions of JARID2-depleted cells with and without LiCl treatment were probed for β-catenin. In control cells, we found that β-catenin was translocated to the nucleus upon differentiation, resulting in an increased level of nuclear β-catenin (Fig. 6c). This increase was not seen in JARID2-depleted cells, which showed reductions in nuclear β-catenin (Fig. 6c). A corresponding increase in cytoplasmic β-catenin could be seen in the JARID2-depleted cells upon differentiation, further supporting the block to β-catenin translocation (Fig. 6d). However, treatment with LiCl restored the translocation of β-catenin to the nucleus in JARID2-depleted cells (Fig. 6c). A corresponding reduction in cytoplasmic β-catenin was also observed (Fig. 6d). We also examined the expression of GSK3β under these conditions and saw no significant change in the expression of GSK3β in JARID2 depleted cells with or without LiCl treatment (Additional file 3: Figure S3).

β-catenin has previously been reported to activate *Myod1* [2], and we had observed that activation of Wnt in JARID2 depleted cells both activated MYOD and enhanced nuclear translocation of β-catenin to the nucleus. We next asked whether β-catenin was bound to the *Myod1* promoter in JARID2-depleted cells following LiCl treatment using ChIP assays. Previous work has shown that β-catenin binds to the DRR of the *Myod1* promoter [2]. We found that β-catenin was bound to the DRR region of the *Myod1* promoter in normal cells upon differentiation and that this binding was lost upon JARID2 depletion (Fig. 6e). However, treatment with LiCl restored the binding of β-catenin to the DRR region of the *Myod1* promoter (Fig. 6e). These effects were specific to the DRR region and were not observed in PRR or CE regions (Fig. 6f, g). Our data show that β-catenin directly activates *Myod1* under control of the JARID2/PRC2 complex.

**Fig. 7:**
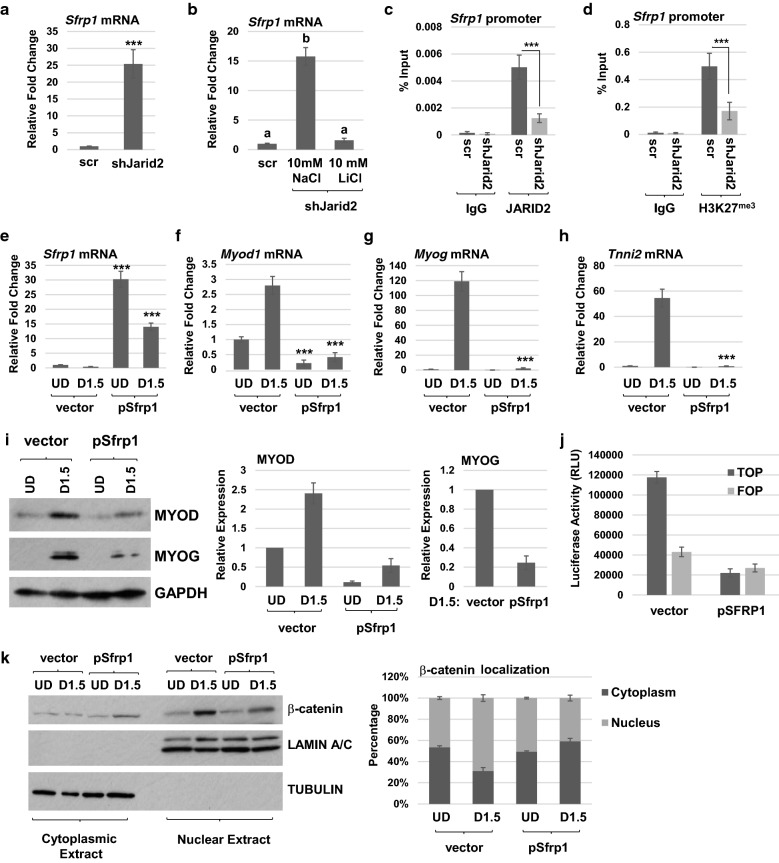
The Wnt antagonist, *Sfrp1*, is directly repressed by JARID2 and the PRC2 complex. **a** *Sfrp1* mRNA is upregulated in JARID2 depleted cells as assayed by qRT-PCR. Error bars are S.E.M. ***p value < 0.001 versus scr. *n* ≥ 3. **b** Activation of canonical Wnt signaling pathway suppresses *Sfrp1* expression. Scr- or shJarid2-expressing cells were differentiated for 2 days and shJarid2 cells were treated with either 10 mM NaCl or 10 mM LiCl while differentiating. mRNA expression of *Sfrp1* was assayed by qRT-PCR. Fold change in expression of mRNA was calculated relative to undifferentiated scramble control. Data are plotted as means with S.E.M. and analyzed with one-way ANOVA followed by Tukey’s HSD post hoc test. Means not sharing same letter are statistically significant (*p* < 0.001). *n* ≥ 3. **c**, **d** JARID2 directly represses *Sfrp1*. Scr or shJarid2 cells were used for ChIP assays using antibodies against JARID2 (**c**), trimethylation of histone 3 lysine 27 (H3K27^me3^) (**d**) and nonspecific antibody (IgG) with primers specific to the *Sfrp1* promoter. Error bars are S.E.M. ***p value < 0.001. *n* ≥ 3. **e**–**h** SFRP1 expression inhibits muscle gene expression. An expression construct for *Sfrp1* (cDNA) was transiently expressed in C2C12 cells and RNA harvested 48 h post-transfection (UD) and following an additional 36 h of differentiation conditions (D1.5). mRNA expression was assayed by qRT-PCR for *Sfrp1* (**e**) *Myod1* (**f**), *Myog* (**g**) and *Tnni2* (**h**). Error bars are S.E.M. ***p value < 0.001 versus time-matched vector. *n* ≥ 3. **i** Inhibition of MYOD and MYOG expression by SFRP1 was confirmed by western blot assays. SFRP1 was transiently expressed in C2C12 cells and protein harvested 48 h post-transfection (UD) and following an additional 36 h of differentiation conditions (D1.5). Data were quantified and normalized to GAPDH (right side of panel). **j** SFRP1 inhibits Wnt-mediated transcriptional activity. Reporter assays were performed using TOP*Flash* and FOP*Flash* luciferase reporters in C2C12 cells transfected with *Sfrp1* (cDNA) or vector control. Growth medium was replaced with differentiation medium 24 h post-transfection, and luciferase activity was detected 48 h post-transfection. Error bars are S.E.M. *n* ≥ 3. **k** SFRP1 inhibits translocation of β-catenin to the nucleus. Nuclear and cytoplasmic fractions of proteins from cells as in **i** were probed for ß-catenin, LAMIN A/C (loading control for nucleus) and tubulin (loading control for cytoplasm). Data were quantitated, normalized to respective loading controls, and plotted as a stacked graph (right side of panel). Error bars are S.E.M. *n* ≥ 3

To identify the molecular mechanism that could explain the block to Wnt signaling in JARID2 depleted cells, we next examined the mRNA expression of two Wnt antagonists, *Sfrp1* and *Nkd1*. We found that *Sfrp1* mRNA was highly upregulated when JARID2 was depleted (Fig. 7a). In contrast, *Nkd1* mRNA showed no upregulation and in fact was downregulated upon JARID2 depletion (Additional file 4: Figure S4 a). We next asked whether activation of Wnt signaling could reverse the upregulation of *Sfrp1*. JARID2 depleted cells were treated with LiCl, and we found that activation of Wnt was sufficient to downregulate *Sfrp1* mRNA (Fig. 7b). To determine whether *Sfrp1* was a direct target of JARID2, we performed ChIP assays and found that JARID2 binds to the *Sfrp1* promoter and depletion of JARID2 causes a loss of this enrichment (Fig. 7c). We also found that H3K27^me3^ was present at the *Sfrp1* promoter in normal cells, indicating that it is normally repressed by the PRC2 complex (Fig. 7d). Upon JARID2 depletion, H3K27^me3^ was reduced on the *Sfrp1* promoter (Fig. 7d), which demonstrates that the promoter methylation of H3K27 is dependent on JARID2. We also found that H3K27^me3^ was present on the *Nkd1* promoter and depletion of JARID2 reduced H3K27^me3^ on this promoter (Additional file 4: Figure S4 b). However, our mRNA expression data show that the loss of H3K27^me3^ on the *Nkd1* promoter is not sufficient for activation of this gene.

To determine whether SRFP1 was responsible for the differentiation block we observed, an expression construct for *Srfp1* (cDNA) was cloned and transiently expressed in C2C12 cells. We confirmed that *Sfrp1* mRNA could be detected in these cells (Fig. 7e). We also found that ectopic expression of SFRP1 was sufficient to inhibit the mRNA expression of *Myod1* (Fig. 7f) and *Myog* (Fig. 7g). RNA expression of a differentiation marker, *Tnni2*, was also strongly inhibited in the presence of SFRP1 (Fig. 7h). The inhibition of MYOD and MYOG by SFRP1 was confirmed at the protein level (Fig. 7i). We also asked whether SFRP1 could inhibit Wnt transcriptional activity using the TOP*Flash*/FOP*Flash* luciferase reporter system and found that transfection with *Sfrp1* (cDNA) robustly inhibited TOP activity (Fig. 7j). We also asked whether SFRP1 was responsible for the block to β-catenin nuclear translocation that we observed in JARID2 depleted cells. We found that ectopic expression of SFRP1 was sufficient to inhibit the nuclear translocation and increase the cytosolic localization of β-catenin (Fig. 7k). These data show that the repression of *Sfrp1* by JARID2 and the PRC2 complex is essential for activation of the canonical Wnt pathway, which normally induces β-catenin translocation to the nucleus for the activation of *Myod1*.

**Fig. 8:**
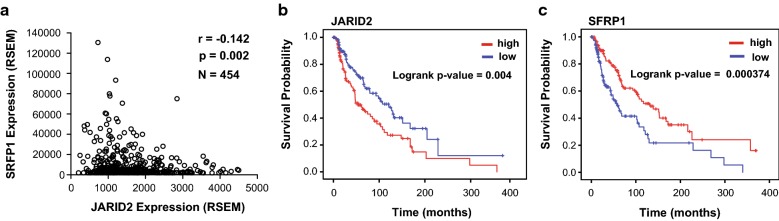
Expression of JARID2 and SFRP1 is negatively correlated. **a** Pearson’s correlation test was performed, and a scatter plot was generated for all patients with expression of *JARID2* on x-axis and corresponding expression of *SFRP1* on y-axis. **b** Patients with higher expression of JARID2 in SKCM have overall poor survival. Kaplan–Meier plot for patients in top 25 percentile (High) and bottom 25 percentile (Low) group of JARID2 and (*n*_High_ = 114, *n*_Low_ = 114). **c** Patients with a lower expression of *SFRP1* in SKCM have poor survival. Kaplan–Meier plot for patients in top 25 percentile (High) and bottom 25 percentile (Low) group of *SFRP1* and (*n*_High_ = 114, n_Low_ = 114)

To determine whether the repression of *Sfrp1* by JARID2 and the PRC2 complex was relevant beyond skeletal muscle, we asked whether the expression of JARID2 was correlated with SFRP1 in the Cancer Genome Atlas (TCGA) data. We first investigated the overall survival correlation between the expression of *JARID2* and *SFRP1* using the OncoLnc tool as previously described [26]. Interestingly, both the genes queried were highly ranked and showed opposite Cox coefficient signs, positive for *JARID2* and negative for *SFRP1*, in several cancer types including skin cutaneous melanoma (SKCM) and breast invasive carcinoma (BRCA). SKCM was chosen for the analysis presented here, but BRCA showed very similar results (data not shown). The expression data (RSEM) and clinical data for all the patients (*N* = 459) from the TCGA database were downloaded using the OncoLnc tool. For the correlation analysis, five of the “extreme outlier” samples were removed (*N* = 454). We found that, as anticipated from our results, the expression of *JARID2* and *SFRP1* was negatively correlated (Fig. 8a). We then sought to understand the survival prognosis between two non-overlapping cohorts of patients divided based on the expression of each gene, with the top 25 percentile defined as “High” and lower 25 percentile defined as “Low” (Additional file 5: Figure S5 a and b). Using Kaplan–Meier analysis, we found that low *JARID2* expression and high *SFRP1* expression were associated with significantly higher overall survival over a 400-month interval (Fig. 8b, c). We further performed correlation analysis between the expression of *JARID2* and *SFRP1* between two groups divided based on *JARID2* expression as used for the survival analysis. We found that the negative correlation between the expression of *JARID2* and *SFRP1* was strengthened (Additional file 5: Figure S5 c). All data obtained from the TCGA database and resulting statistical analysis are shown in Additional file 6: File S1.

**Fig. 9:**
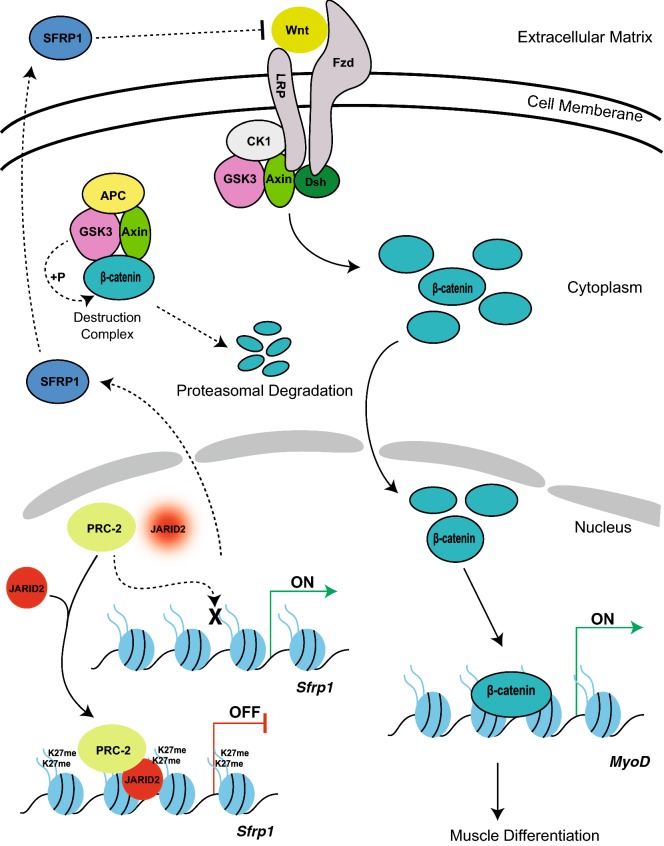
Schematic model for Wnt modulation by JARID2 in a PRC2 complex-dependent manner. JARID2 directs the PRC2 complex to repress *Sfrp1*, a secreted negative regulator of Wnt. Repression of *Sfrp1* allows Wnt-activated β-catenin to activate *Myod1*, which is required for myogenesis. Dashed arrowheads and T-bars represent the events following depletion of JARID2 (represented by fading oval)

Taken together, our results establish that JARID2 and the PRC2 complex are required for skeletal muscle differentiation by directly repressing the Wnt antagonist, *Sfrp1*. Repression of *Sfrp1* allows Wnt signaling to activate *Myod1* for myogenic differentiation to proceed (Fig. 9). The repression of *Sfrp1* by JARID2 and the PRC2 complex clearly extends beyond skeletal muscle and has important implications for development, adult homeostasis and disease.

## Discussion

We show here that JARID2 and the PRC2 complex are required to initiate skeletal muscle differentiation by modulating the Wnt signaling pathway that is required to activate *Myod1*. The direct repression of *Sfrp1* by JARID2 allows Wnt signaling to promote myogenesis. The role of Wnt signaling during myogenesis is complex, but it is well established that Wnt signaling is essential for embryonic muscle development and adult muscle regeneration (reviewed in [1]). Canonical Wnt signaling has been shown to be required for the differentiation of myoblasts [27]. The importance of canonical Wnt signaling in myogenesis has been demonstrated through series of loss- and gain-of-function assays that showed loss of β-catenin delayed differentiation, while constitutive overexpression of β-catenin results in precocious differentiation [28]. However, the molecular pathways controlling this process have not been fully characterized. Recent work has shown that the methyltransferase, SETD7, enhances skeletal muscle differentiation by priming β-catenin for nuclear import, thus enabling Wnt signaling [29]. Wnt signaling controls myogenic differentiation in part by regulating expression of the MRFs, including MYOD and MYF5 [2, 30–32]. However, the molecular mechanisms by which the Wnt pathway controls MRF expression have remained to be elucidated.

Our data provide more evidence of the essential role that Wnt signaling plays in myogenesis, but also expands the understanding of the roles of JARID2 and the PRC2 complex in skeletal muscle. The PRC2 complex has been shown to be required for satellite cell proliferation [8], but our results show that this complex is also critical for the transition to the terminal differentiation stage of myogenesis. Importantly, we also show here that JARID2 is required to guide the PRC2 complex to many of the previously described gene targets of the PRC2 complex, including *Myog* [18]. The impaired expression of MYOD and MYOG observed upon depletion of JARID2 or EZH2 was highly surprising based on previous studies. We have shown that depletion of JARID2 modestly relieves the potent suppression of *Myog* mRNA expression by exogenous CIITA [11]. Enhanced EZH2 expression has been shown to inhibit differentiation [9]. In this study, an siRNA approach with EZH2 led to a decrease in promoter methylation of differentiation-specific genes. Significantly, while decreases in promoter methylation at genes such as *Mck* were observed upon EZH2 depletion, a corresponding increase in protein expression was not observed, consistent with our work. Other studies have also observed the anticipated increased expression of myogenin and downstream targets upon Suz12 depletion, but this study utilized a transient siRNA approach [18]. We show here that a transient approach using the shRNA constructs characterized here also results in a transient upregulation of *Myog* mRNA, indicating that temporal and overall level of EZH2 depletion will lead to differential effects, complicating any comparison of the results. The data suggest that the level of EZH2 is precisely controlled in skeletal muscle cells and positive or negative alterations to that level leads to deleterious effects and differential experimental outcomes. A conditional ablation of *Ezh2* in satellite cells using a *Pax7*-Cre driver showed an increase in the MYOG-positive satellite cell pool, indicating precocious differentiation, but a delay in MYOG-positive cells following injury [8]. Other studies have shown that siRNA against EZH2 in isolated satellite cells leads to a downregulation of myogenin and inhibition of EZH2 in these cells leads to decreases in downstream targets such as MCK [33]. These results highlight the complexity of the role of EZH2 in skeletal muscle. Additional studies have shown that depletion of *JARID2* mRNA [34] or inhibition of EZH2 [35] led to increases in *MYOG* mRNA and differentiation-specific gene expression in rhabdomyosarcoma (RMS) cell lines. Any comparison with RMS cell lines is complicated by the fact that RMS cells are shown to overexpress JARID2 and PRC2 proteins and, yet, highly express MYOD and MYOG and still fail to differentiate. A repressive basic helix-loop-helix factors, musculin (MSC), antagonize the activity of MYOD in myoblasts and in rhabdomyosarcoma [36, 37]. Additionally, Wnt signaling is known to be deregulated in RMS cells [38, 39], which is likely to contribute to the significant differences we observe in C2C12 cells. Intriguingly, SFRP3 has been shown to be required for PAX3-FOXO1-mediated tumorigenesis [40], again highlighting the deregulation of Wnt signaling in these cells.

The molecular explanation of our surprising result that JARID2 and EZH2 were required to activate MYOD in normal cells was suggested by studies using high-throughput techniques that showed that JARID2 and the polycomb (PcG) group of proteins have a large number of overlapping targets in embryonic stem (ES) cells, which included several Wnt signaling pathway genes and suggested a possible regulation of the Wnt signaling pathway by JARID2 [13, 41]. Additionally, the loss of JARID2 in murine ES cells was shown to result in the constitutive overexpression of *Nanog* mRNA and a reduction in the activity of β-catenin along with a downregulation of Wnt/PCP activity [12]. In this study, a panel of Wnt antagonists, including *Sfrp1*, was found to be de-repressed, although no change in H3K27^me3^ on the *Sfrp1* promoter was observed in *Jarid2* null cells, suggesting that the JARID2 regulation of *Sfrp1* was independent of PRC2 in mES cells [12]. Interestingly, one of the Wnt antagonists included in the previous study, *NKD1*, was shown to be directly regulated by the PRC2 complex in hepatocellular carcinoma [42]. In our study, while we observed a decrease in H3K27^me3^ status on the promoter of *Nkd1* upon depletion of JARID2, we saw no upregulation in mRNA expression. However, our identification of *Sfrp1* as a direct target of JARID2 is highly significant as we establish here that not only is *Sfrp1* a direct target of JARID2 and the PRC2 complex in skeletal muscle cells, but that SFRP1 is sufficient to recapitulate the Wnt signaling block observed in JARID2 depleted cells.

Our work supports a growing body of evidence showing an essential role for JARID2 during development. JARID2 has previously been shown to be required for heart development [22]. However, in heart development, JARID2 functions in association with H3K9 methyltransferases including SETD1, G9a and GLP [22, 43–45]. In contrast, at the majority of targets we studied in C2C12 cells, we did not see any significant changes in H3K9^me^ status upon JARID2 depletion. Instead, we observed a significant change in H3K27^me3^ status at each of these genes, indicating a PRC2-dependent function of JARID2. This is in agreement with previous studies that have shown the role of JARID2 in targeting the PRC2 complex to target genes [11, 13, 19, 41]. However, earlier studies with ES cells showed that loss of *Jarid2* delayed ES cell differentiation, while loss of PRC2 enhanced differentiation [19]. This was attributed to the repressive role of JARID2 on PRC2 complex activity. In our study, we find that depletion of JARID2 or EZH2 inhibits skeletal muscle differentiation through co-regulation of *Sfrp1* and the downstream regulation of the MRFs required for differentiation. Our data do not exclude a yet uncharacterized role of JARID2 in guiding H3K9 methyltransferases in skeletal muscle, and this remains to be further explored in future studies.

The data showing that JARID2 and SFRP1 are inversely correlated in melanoma are intriguing and suggest that JARID2 and PRC2’s modulation of Wnt signaling is highly significant for many cellular systems. EZH2 is known to be highly expressed in many cancers (reviewed in [46]), and elevated levels of JARID2 have been implicated in several cancers as well [34, 47–49]. Deregulated Wnt signaling has also shown to be correlated with poor survival in many cancer studies (reviewed in [50]). In this study, we provide evidence on the direct relationship between epigenetic modifiers, JARID2 and the PRC2 complex, and a canonical Wnt signaling regulator, SFRP1, that are significantly correlated in cancer patients and significantly associated with the lifespan and overall survival of these patients. The correlation further strengthens the molecular mechanism we discovered using an in vitro cell system. In addition to SKCM, multivariate Cox regression analysis using OncoLnc showed that overall survival of the patients was also correlated in four other cancer types in a similar fashion as shown for SKCM, including breast invasive carcinoma (BRCA). Thus, the relationship discovered in this study between JARID2 and the PRC2 complex and the Wnt signaling pathway goes beyond myogenesis. Uncovering how JARID2 and SFRP1 modulate cancer progress and patient survival in these cancers will be an important future direction of this study. Several therapeutic agents targeting the Wnt pathway have entered clinical trials [51], and EZH2 inhibitors are also entering clinical trials [52]. Wnt and EZH2 targeting agents have shown clinical promise, but it has been noted that caution must be taken with these agents as both pathways are also indispensable for development processes and adult homeostasis. Precisely understanding the molecular mechanisms utilized in both normal and cancer cells will be required to effectively employ these novel therapeutic approaches.

## Conclusions

We show that, like EZH2, JARID2 is expressed in proliferating myoblasts, but downregulated upon differentiation. JARID2 guides the PRC2 complex to many of the known PRC2 targets in skeletal muscle. We also reveal the unexpected finding that JARID2 and PRC2 are required to initiate skeletal muscle differentiation by modulating Wnt signaling through the direct repression of Wnt antagonists.

## Methods

### Cell culture

C2C12 cells (ATCC) were grown in Dulbecco’s modified Eagle medium (DMEM) supplemented with 10% fetal bovine serum (HyClone) according to standard protocols. Proliferating C2C12 myoblasts were grown in DMEM supplemented with 10% fetal bovine serum (HyClone). To induce differentiation of C2C12 myoblasts into myotubes, cells were grown to 90% confluence and the media was switched to DMEM supplemented with 2% horse serum (HyClone). C2C12 cells were grown in differentiation medium for the number of days indicated in each experiment. Primary myoblasts were isolated according to standard protocols [53].

### shRNA knock down

Jarid2 was depleted with shRNA constructs (pLOK.1, Sigma) as previously described [11]. Ezh2 knock down lines were constructed with shRNA constructs designed by the RNAi Consortium in the pLOK.1 plasmid (Open Biosystems). Three constructs targeting murine *Jarid2* and *Ezh2* mRNA, and one scrambled control were linearized, transfected into C2C12 cells, and selected with puromycin (2 μg/ml). Individual clones were selected and propagated.

### Cell transfections

C2C12 cells were transfected with TurboFect (Fisher Scientific) according to manufacturer’s protocol. The plasmid pEMSV-MyoD (provided by Andrew Lassar, Harvard Medical School) was used for expressing MYOD. Active Wnt3A-V5 was a gift from Xi He (Addgene plasmid #43810) [25]. All transfections were performed in three technical replicates, and a minimum of three biological replicates were performed for each experiment.

### Cloning

To clone murine genes, total RNA from C2C12 cells was extracted with Trizol (Invitrogen) and used to generate cDNA through the use of the Superscript III First Strand Synthesis System for RT-PCR kit with oligo dT primers (Invitrogen). Murine SFRP1 was amplified from cDNA generated from undifferentiated C2C12 cells using primers corresponding to the coding regions with engineered restriction sites. Primers are described in Additional file 7: Table S1. The resulting PCR product was cloned using restriction enzymes NotI and XhoI and the pCMV-tag1 cloning vector (Agilent). All resulting clones were confirmed by sequencing.

### TOPFlash/FOPFLash luciferase assays

C2C12 cells were transfected with TurboFect (Fisher Scientific) according to standard protocols. M50 Super 8x TOPFlash (Addgene plasmid # 12456) and M51 Super 8x FOPFlash (TOPFlash mutant) (Addgene plasmid # 12457) were gifts from Randall Moon [54]. Luciferase activity was determined using the Dual-Luciferase Reporter Assay System (Promega). C2C12 cells were seeded at a density of 2 × 10^3^ cell per well in 96-well plates and transfected with 0.4 μg of DNA. Transfections were normalized to Renilla luciferase. Transfections were performed in triplicate, and all data sets were repeated three times.

### Western blot analysis

Cell extracts were made by lysing phosphate-buffered saline (PBS)-washed cell pellets in radio-immunoprecipitation assay buffer (RIPA) supplemented with protease inhibitors (Complete protease inhibitor, Roche Diagnostics). Following incubation on ice, clear lysates were obtained by centrifugation. Protein concentrations were determined by Bradford’s assay (Bio-Rad). For each sample, 30 µg of protein was loaded on each gel. Proteins were transferred onto a PVDF membrane using a tank blotter (Bio-Rad). The membranes were then blocked with 5% nonfat milk in 1X Tris-buffered saline plus Tween 20 (TBST) and incubated with primary antibody overnight at 4 °C. Membranes were then washed with 1X TBST and incubated with the corresponding secondary antibody. Membranes were again washed with 1X TBST, incubated with chemiluminescent substrate according to manufacturer’s protocol (SuperSignal, Pierce) and visualized by autoradiography. The antibodies used include anti-Jarid2 (Cell Signaling), anti-Ezh2 (Cell Signaling), anti-myogenin (F5D, Developmental Studies Hybridoma Bank (DSHB)), anti-MyoD (C-17, Santa Cruz Biotechnology (SCBT)), anti-β-catenin (E5, SCBT), anti-MHC (MF-20, DSHB), anti-Lamin A/C (E-1, SCBT), anti-TBP (Cell Signaling), anti-GAPDH (Millipore) and anti-GSK3β (0011-A, SCBT). Densitometry to quantify protein expression levels was performed with ImageJ software (NCBI) on at least three independent experiments. Representative autoradiography films are shown.

### Quantitative real-time PCR

RNA was isolated from cells by Trizol extractions (Invitrogen). Following treatment with DNase (Promega), two micrograms of total RNA was reverse transcribed with MultiScribe™ MuLV reverse transcriptase (Applied Biosystems). cDNA equivalent to 40 ng was used for quantitative polymerase chain reaction (qPCR) amplification (Applied Biosystems) with SYBR green PCR master mix (Applied Biosystems). Samples in which no reverse transcriptase was added (no RT) were included for each RNA sample. qPCR data were calculated using the comparative Ct method (Applied Biosystems). Standard deviations from the mean of the [Δ] *Ct* values were calculated from three independent RNA samples. Primers are described in Additional file 7: Table S1. Where possible, intron-spanning primers were used. All quantitative PCR was performed in three technical replicates and three independent RNA samples representing biological replicates were assayed for each time point. For measurements of relative gene expression, a fold change was calculated for each sample pair and then normalized to the fold change observed at HPRT and/or 18S rRNA.

### Chromatin immunoprecipitation assays

ChIP assays were performed as described previously [55]. The following antibodies were used: anti-Jarid2 (Cell Signaling), anti-H3K27^me3^ (Cell Signaling), anti-pan methyl H3K9 (D54, Cell Signaling) and anti-β-catenin (E-5, SCBT). Rabbit IgG (SCBT) was used as a nonspecific control. Primers are described in Additional file 7: Table S1. The real-time PCR was performed in three technical replicates. The results are represented as percentage of IP over input signal (% Input). All ChIP assays shown are representative of at least three individual biological replicate experiments. Standard error from the mean was calculated and plotted as error bar.

### TCGA (the cancer genome atlas) data analysis

The “OncoLnc” tool was used to download patient information on skin cutaneous melanoma (SKCM) from The Cancer Genome Atlas (TCGA) database: http://cancergenome.nih.gov/, as described [26]. Using Kaplan–Meier analysis, survival plots were generated for non-overlapping top 25 percentile (high) against bottom 25 percentile (low) patient groups divided according to the mRNA expression level of *JARID2* and *SFRP1*. The expression data downloaded from TCGA for all the patients were used for Pearson’s correlation test between *JARID2* and *SFRP1*, and scatterplots were generated. Student’s t test was performed between non-overlapping high- and low-group patients for *JARID2* and *SFRP1* expression level and was plotted as box plots. All the statistical analysis and graphs were made using SPSS Statistic 24.0.0 software.

### Statistics

Data are presented as means ± standard errors (S.E.). Statistical comparisons were performed using unpaired two-tailed Student’s *t* tests or one-way ANOVA followed by Tukey’s HSD (honest significant difference) post hoc test. For one-way ANOVA analysis, all means not sharing the same letter were statistically significant. Probability value of < 0.05 was taken to indicate significance.

## Additional files

**Additional file 1: Figure S1**. *Myod1* and *Myog* are direct targets of the PRC2 complex. **a**–**c** *Myod1* is a direct target of JARID2 and the PRC2 complex. ChIP assays using antibodies against trimethylation of lysine 27 of histone 3 (H3K27^me3^) and a nonspecific antibody (IgG) were performed on C2C12 cells stably expressing scr and shJarid2. Primers spanning three regulatory regions of the *Myod1* promoter were used: core enhancer (CE) (**a**), distal regulatory region(DRR) (**b**) and proximal regulatory region (PRR) (**c**). **d**, **e**. Myogenin is a direct target of JARID2 and the PRC2 complex. Cells from (**a**) were differentiated for 2 days and subjected to ChIP assays performed and analyzed as in (**a**). Primers spanning two different regions of the myogenin promoter, 1.5-kb upstream of the transcription start site (1.5 kb) (**d**) and proximal promoter (*Myog E1,2*) (**e**) were used. n.s. is not statistically significant. F. H3K27^me3^ at the *HoxB7* promoter is unaffected by depletion of JARID2. ChIP assays were performed and analyzed as in E.G. H3K9 methylation of *Myod1* is not dependent on JARID2. ChIP assays using antibodies against trimethylation of lysine 9 of histone 3 (H3K9^me^) and a nonspecific antibody (IgG) were performed on C2C12 cells stably expressing scr and shJarid2. Primers spanning three regulatory regions of the *Myod1* promoter were used: core enhancer (CE) (**g**), distal regulatory region (DRR) (**h**), and proximal regulatory region (PRR) (**i**). **j** H3K9 methylation is not observed on the *Myog* proximal promoter. ChIP assays were performed as in **g**. **k** H3K9 methylation is reduced on the upstream 1.5-kb region of *Myog* when JARID2 is depleted. ChIP assays preformed as in G. Error bars are S.E.M. **p value < 0.01 and ***p value < 0.001. *n* ≥ 4.

**Additional file 2: Figure S2**. Exogenous expression of MYOD restores myogenin and late muscle gene expression in JARID2 depleted C2C12 cells. A. Plasmids expressing *Myod1* (cDNA) or empty vector were transiently transfected in C2C12 cells stably expressing shRNA against *Jarid2* mRNA. Total RNA was extracted 48 h post-transfection (UD) or 96 h post-transfection with 48 h in low serum media (D2). mRNA expression of *Myod1* was assayed by qRT-PCR. Relative mRNA expression was calculated relative to the vector UD sample. Error bars are S.E.M. ***p value < 0.001 versus time matched vector. *n* ≥ 3. **b** Myogenin expression is restored upon exogenous expression of MYOD. mRNA expression of myogenin was analyzed by qRT-PCR as in **a**. **c** Late muscle gene expression is induced upon transient MYOD expression. Cells from (**a**) were used to assay for mRNA expression of myosin light chain (*Mylpf*) by qRT-PCR and analyzed. Error bars are S.E.M. ***p value < 0.001 versus time-matched vector. *n* ≥ 3.

**Additional file 3: Figure S3**. Expression of GSK3β is unaltered in Jarid2 depletions irrespective of Wnt activation. C2C12 cells stably expressing scramble shRNA (scr) or *Jarid2* mRNA-specific shRNA (shJarid2) were grown to confluency in high serum (UD) and switched to differentiation conditions for 2 days (D2). shJarid2 cells were treated with either 10 mM NaCl or 10 mM LiCl for 2 days in differentiation conditions as indicated. Total cell extracts were probed as indicated. Gels were quantified and normalized to respective loading controls (lower panel). Relative expression was calculated relative to scr UD sample and plotted as bar graphs. Error bars are S.E.M. *n* ≥ 3.

**Additional file 4: Figure S4**. *Nkd1* is not activated by JARID2 depletion. **a** *Nkd1* mRNA is downregulated in JARID2 depleted cells as assayed by qRT-PCR. Error bars are S.E.M. **p < 0.001 versus scr. *n* ≥ 3. **b** The *Nkd1* promoter is methylated in a JARID2 dependent manner, scr or shJarid2 cells were used for ChIP assays using antibodies against trimethylation of histone 3 lysine 27 (H3 K27^me3^) and nonspecific antibody (IgG) with primers specific to the *Nkd1* promoter. Error bars are S.E.M. *n* ≥ 3.

**Additional file 5: Figure S5**. Expression of JARID2 and SFRP1 is better correlated in groups divided based on JARID2 expression. **a**, **b** Box plot representing the expression of *JARID2* mRNA (**a**) and *SFRP1*mRNA (**b**) in between lower 25 percentile and upper 25th percentile, respectively (*p* < 0.001). **c** Pearson’s correlation test was performed between the expression of *JARID2* mRNA and *SFRP1* mRNA after splitting all patients into two categories, top 25 percentile and bottom 25 percentile, based on expression of *JARID2* mRNA.

**Additional file 6: File S1**. TCGA data analysis used in study.

**Additional file 7: Table S1**. Oligonucleotides used in study.

## Abbreviations

Wnt: wingless-related integration site
H3K27: histone H3 lysine 27
H3K9: histone H3 lysine 9
mRNA: messenger RNA
cDNA: complementary DNA
shRNA: short/small hairpin RNA
siRNA: short interfering RNA
