# Supplementary figures and images for: JARID2 and the PRC2 complex regulate skeletal muscle differentiation through regulation of canonical Wnt signaling
Source: Epigenetics Chromatin. 2018 Aug 17;11:46. doi: 10.1186/s13072-018-0217-x (PMC6097338; doi:10.1186/s13072-018-0217-x)

Supplemental Figure 1

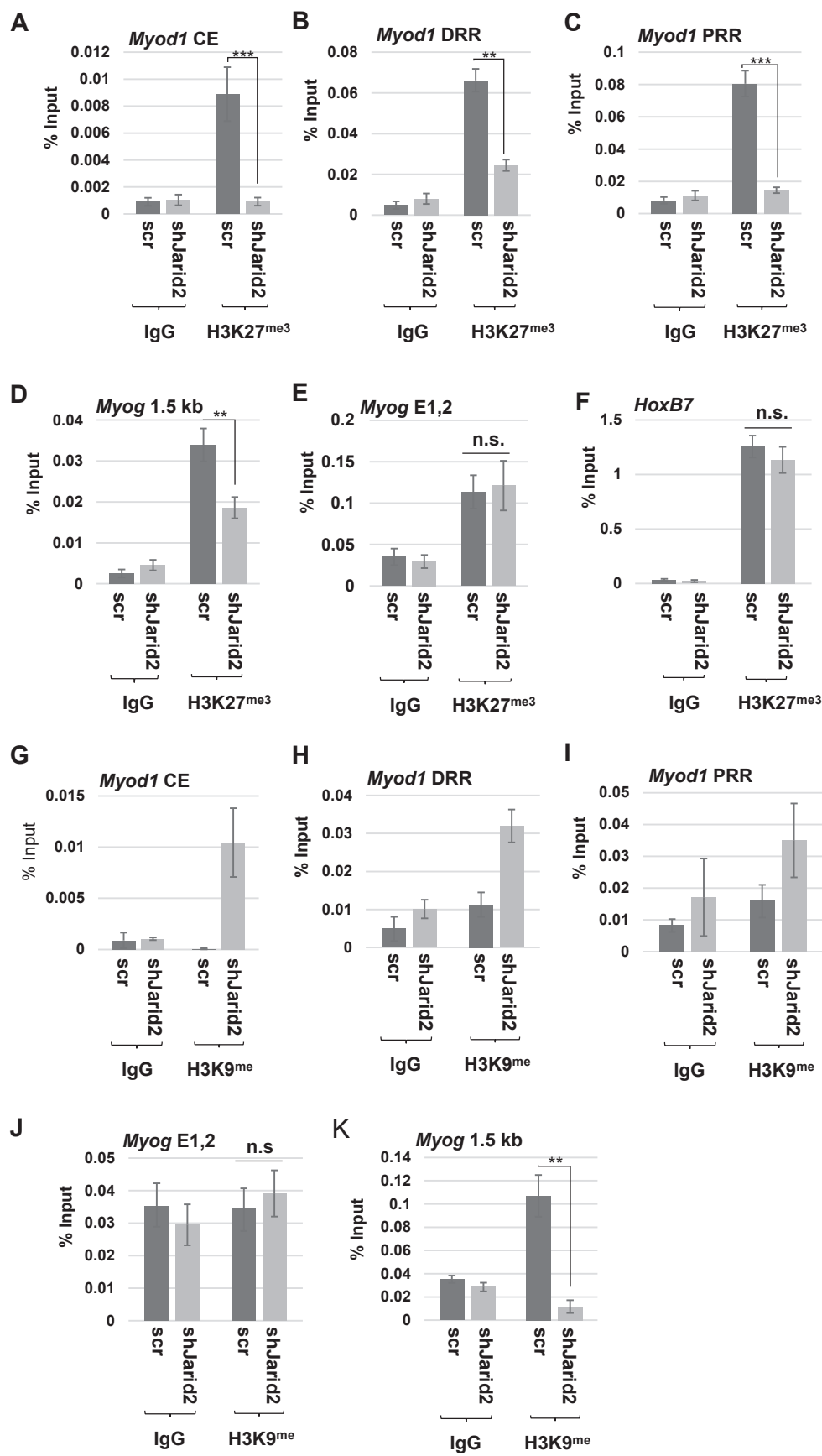

Supplement: Supplementary file 1 — Additional file 1: Figure S1. Myod1 and Myog are direct targets of the PRC2 complex. a–c Myod1 is a direct target of JARID2 and the PRC2 complex. ChIP assays using antibodies against trimethylation of lysine 27 of histone 3 (H3K27me3) and a nonspecific antibody (IgG) were performed on C2C12 cells stably expressing scr and shJarid2. Primers spanning three regulatory regions of the Myod1 promoter were used: core enhancer (CE) (a), distal regulatory region(DRR) (b) and proximal regulatory region (PRR) (c). d, e. Myogenin is a direct target of JARID2 and the PRC2 complex. Cells from (a) were differentiated for 2 days and subjected to ChIP assays performed and analyzed as in (a). Primers spanning two different regions of the myogenin promoter, 1.5-kb upstream of the transcription start site (1.5 kb) (d) and proximal promoter (Myog E1,2) (e) were used. n.s. is not statistically significant. F. H3K27me3 at the HoxB7 promoter is unaffected by depletion of JARID2. ChIP assays were performed and analyzed as in E.G. H3K9 methylation of Myod1 is not dependent on JARID2. ChIP assays using antibodies against trimethylation of lysine 9 of histone 3 (H3K9me) and a nonspecific antibody (IgG) were performed on C2C12 cells stably expressing scr and shJarid2. Primers spanning three regulatory regions of the Myod1 promoter were used: core enhancer (CE) (g), distal regulatory region (DRR) (h), and proximal regulatory region (PRR) (i). j H3K9 methylation is not observed on the Myog proximal promoter. ChIP assays were performed as in g. k H3K9 methylation is reduced on the upstream 1.5-kb region of Myog when JARID2 is depleted. ChIP assays preformed as in G. Error bars are S.E.M. **p value < 0.01 and ***p value < 0.001. n ≥ 4. [file 13072_2018_217_MOESM1_ESM.pdf]

Supplemental Figure 2

A

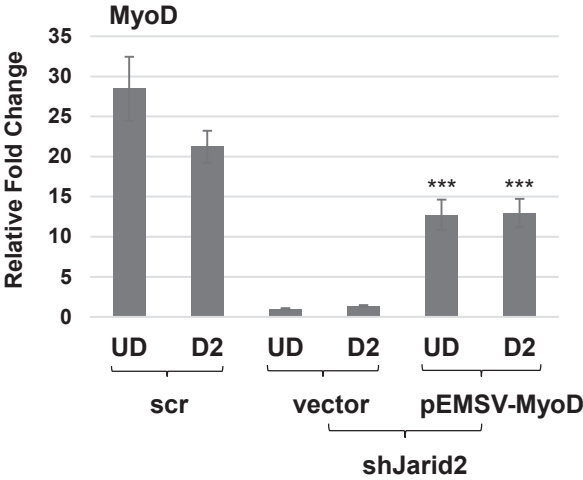

B

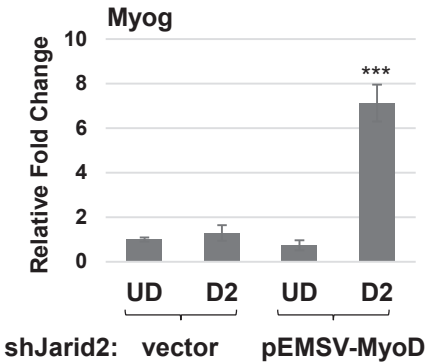

C

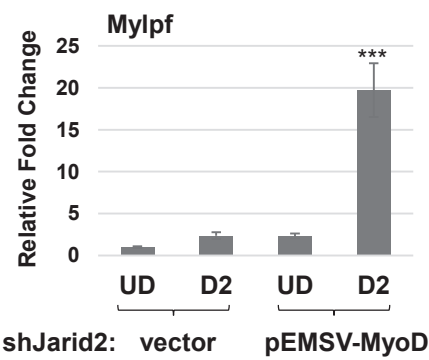

Supplement: Supplementary file 2 — Additional file 2: Figure S2. Exogenous expression of MYOD restores myogenin and late muscle gene expression in JARID2 depleted C2C12 cells. A. Plasmids expressing Myod1 (cDNA) or empty vector were transiently transfected in C2C12 cells stably expressing shRNA against Jarid2 mRNA. Total RNA was extracted 48 h post-transfection (UD) or 96 h post-transfection with 48 h in low serum media (D2). mRNA expression of Myod1 was assayed by qRT-PCR. Relative mRNA expression was calculated relative to the vector UD sample. Error bars are S.E.M. ***p value < 0.001 versus time matched vector. n ≥ 3. b Myogenin expression is restored upon exogenous expression of MYOD. mRNA expression of myogenin was analyzed by qRT-PCR as in a. c Late muscle gene expression is induced upon transient MYOD expression. Cells from (a) were used to assay for mRNA expression of myosin light chain (Mylpf) by qRT-PCR and analyzed. Error bars are S.E.M. ***p value < 0.001 versus time-matched vector. n ≥ 3. [file 13072_2018_217_MOESM2_ESM.pdf]

Supplemental Figure 3

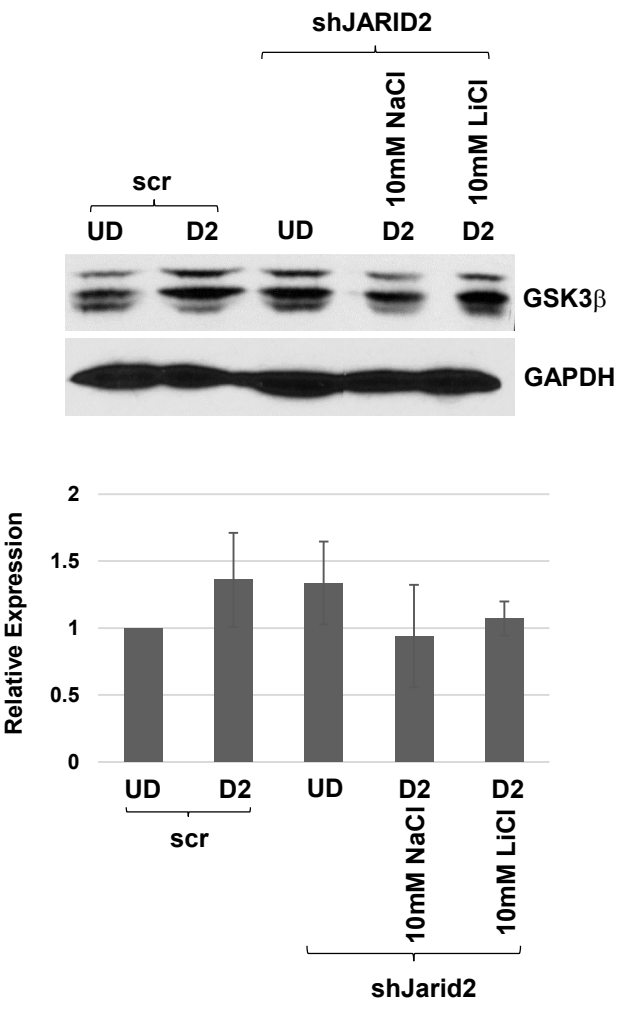

Supplement: Supplementary file 3 — Additional file 3: Figure S3. Expression of GSK3β is unaltered in Jarid2 depletions irrespective of Wnt activation. C2C12 cells stably expressing scramble shRNA (scr) or Jarid2 mRNA-specific shRNA (shJarid2) were grown to confluency in high serum (UD) and switched to differentiation conditions for 2 days (D2). shJarid2 cells were treated with either 10 mM NaCl or 10 mM LiCl for 2 days in differentiation conditions as indicated. Total cell extracts were probed as indicated. Gels were quantified and normalized to respective loading controls (lower panel). Relative expression was calculated relative to scr UD sample and plotted as bar graphs. Error bars are S.E.M. n ≥ 3. [file 13072_2018_217_MOESM3_ESM.pdf]

Supplemental Figure 4

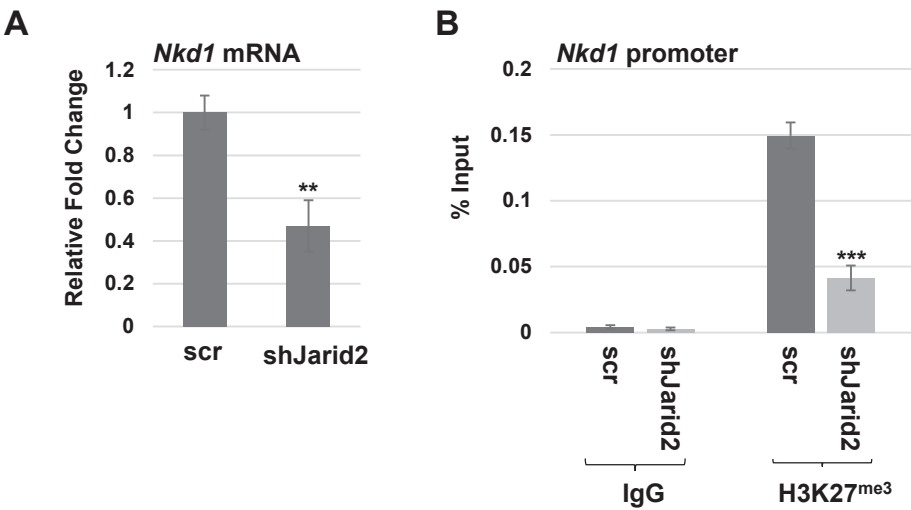

Supplement: Supplementary file 4 — Additional file 4: Figure S4. Nkd1 is not activated by JARID2 depletion. a Nkd1 mRNA is downregulated in JARID2 depleted cells as assayed by qRT-PCR. Error bars are S.E.M. **p < 0.001 versus scr. n ≥ 3. b The Nkd1 promoter is methylated in a JARID2 dependent manner, scr or shJarid2 cells were used for ChIP assays using antibodies against trimethylation of histone 3 lysine 27 (H3 K27me3) and nonspecific antibody (IgG) with primers specific to the Nkd1 promoter. Error bars are S.E.M. n ≥ 3. [file 13072_2018_217_MOESM4_ESM.pdf]

Supplemental Figure 5

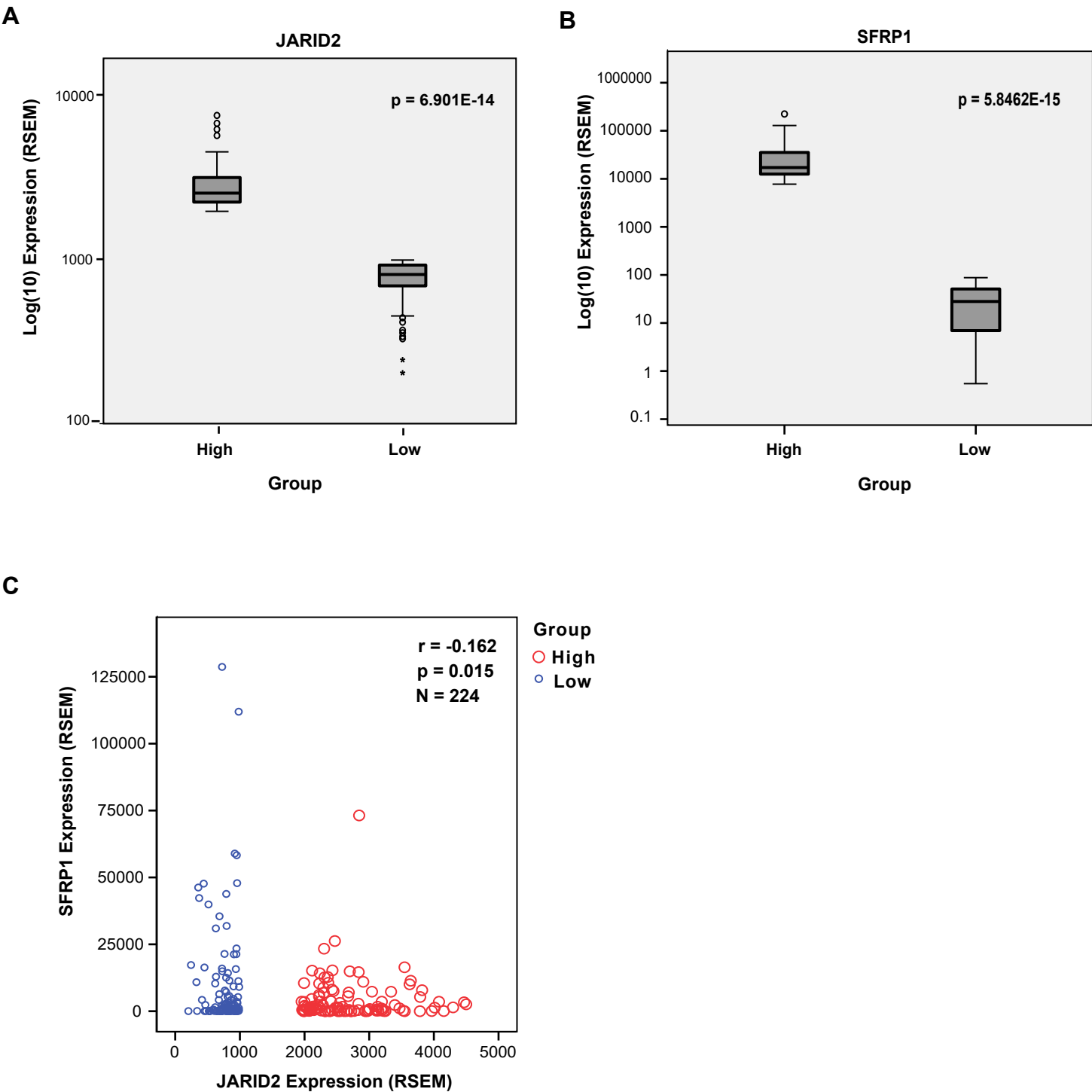

Supplement: Supplementary file 5 — Additional file 5: Figure S5. Expression of JARID2 and SFRP1 is better correlated in groups divided based on JARID2 expression. a, b Box plot representing the expression of JARID2 mRNA (a) and SFRP1mRNA (b) in between lower 25 percentile and upper 25th percentile, respectively (p < 0.001). c Pearson’s correlation test was performed between the expression of JARID2 mRNA and SFRP1 mRNA after splitting all patients into two categories, top 25 percentile and bottom 25 percentile, based on expression of JARID2 mRNA. [file 13072_2018_217_MOESM5_ESM.pdf]
