# Supplementary material for: JARID2 and the PRC2 complex regulate skeletal muscle differentiation through regulation of canonical Wnt signaling
Source: Epigenetics Chromatin. 2018 Aug 17;11:46. doi: 10.1186/s13072-018-0217-x (PMC6097338; doi:10.1186/s13072-018-0217-x)
Supplement: Supplementary file 7 — Additional file 7: Table S1. Oligonucleotides used in study. [file 13072_2018_217_MOESM7_ESM.docx]

**Primers**

Cloning

SFRP1 m BamHI F 5’ atggatccAGCAACATGGGCGTCG 3’

SFRP1 m XhoI R 5’ cgctcgagcttaaaaacagactggaaggtgg 3’

ChIP

MyoD CE F 5’GGCATTTATGGGTCTTCCTAT 3’

MyoD CE R 5’CTAGGCCTGAGCTAGAGAAACC 3’

MyoD DRR F 5’CATCCTCCAGTCCTTCAGC 3’

MyoD DRR R 5’ ggaatgttggtctggctagg 3’

MyoD PRR F 5’ GCTGCACCAGATAGCCAAG 3’

MyoD PRR R 5’ CCTCAAGCCAATAGGAGTGTAGT 3’

mmMyog -1.5 F 5’ CTCCCCCACCTGACATTCTA 3’

mmMyog -1.5 R 5’ GGCTGGCCTCTTCTATTCCT 3’

myog E12 m F 5’ ggaatcacatgtaatccactgg 3’

myog E12 m R 5’ tcacaccaactgctgggt 3’

SFRP1 m promII F 5’ GACGTCGCCGAGCAACAT 3’

SFRP1 m promII R 5’cggactggaagctcacgtag 3’

mRNA expression

Myog F 5’ GACCTGATGGAGCTGTATGAG 3’

Myog R 5’ CTGAAGGTGGACAGGAAGG 3’

MyoD F 5’ GCCGGTGTGCATTCCAA 3’

MyoD R 5’ CACTCCGGAACCCCAACAG 3’

Tnni2 F 5’ GCCGCCGAGAATCTGAGA 3’

Tnni2 R 5’ GACATGGAGCCTGGGATGTG 3’

Mylpf F 5’ GGCTGCCGGGGCAGGACTAT 3’

Mylpf R 5’ CGGCCCATGGCTGCAAAGGT 3’

Axin2 m h F 5’ GGCTATGTCTTTGCACCAGC 3’

Axin2 m h R 5’ tgaggtagagacacttggcca 3’

CTNNB1 m h F 5’ ccatctgtgctcttcgtcat 3’

CTNNB1 m h R 5’ CCTTTATCAGAGGCCAGTGG 3’

NKD1 m/h F 5’ CAGCTTGCTGCATACCATCT 3’

NKD1 m/h R 5’ GGACGCTCCTCTTACTCTGG 3’

SFRP1 m/h F 5’ ACGTGGGCTACAAGAAGATG 3’

SFRP1 m/h R 5’ CCAGTAGAAGCCGAAGAACTG 3’
